# Whole-Genome Sequencing of Multidrug-Resistant Campylobacter fetus subsp. *fetus* NWU_ED24, Isolated from a Bovine Sheath Wash Sample

**DOI:** 10.1128/MRA.00852-20

**Published:** 2020-10-01

**Authors:** Mpinda Edoaurd Tshipamba, Kazeem Adekunle Alayande, Ngoma Lubanza, Mulunda Mwanza

**Affiliations:** a Department of Animal Health, School of Agriculture, Faculty of Agriculture, Science, and Technology, Mafikeng Campus, North-West University, Mmabatho, South Africa; b Department of Microbiology, North-West University, Mmabatho, South Africa; University of Maryland School of Medicine

## Abstract

Campylobacter fetus subsp. *fetus* is an opportunistic human pathogen that is frequently identified as a cause of intestinal infections as well as bloodstream infections. This bacterium is well known to cause spontaneous abortions in sheep and cows. The strain reported in this study was isolated from a preputial wash sample from a bull in South Africa.

## ANNOUNCEMENT

Campylobacter fetus subsp. *venerealis* and C. fetus subsp. *fetus* are associated with infections in mammals (1, 2), while C. fetus subsp. *testudinum* is isolated primarily from reptiles (3). Reptile-associated C. fetus subsp. *testudinum* is genetically distant from mammal-associated C. fetus subsp. *venerealis* and C. fetus subsp. *fetus*, which have a high level of genetic relatedness (4, 5). Campylobacter fetus subsp. *fetus* NWU_ED24 was isolated from a preputial wash sample and cultured on tryptose blood agar base CM0233 (Oxoid, United Kingdom) mixed with 7% sheep blood and supplemented with *Campylobacter* selective supplement (Skirrow) SR0068E (Oxoid). The plates were incubated at 37°C for 72 h in a 2.5-liter anaerobic jar (Oxoid) equipped with a CampyGen CN0025A sachet (Oxoid) to create microaerophilic conditions (6). Colonies from pure cultures of C. fetus subsp. *fetus* NWU_ED24 on the tryptose blood agar were carefully scraped and washed twice in phosphate-buffered saline (pH 7). Genomic DNA was extracted from the washed cells using a Zymo Research (USA) kit (D6005). The 16S rRNA region was amplified, and the PCR amplicon was sequenced. The nucleotides were aligned with sequences in the NCBI database using the BLAST algorithm to confirm the identity of the isolate (7).

The extracted genomic DNA samples were fragmented using an enzymatic approach (NEBNext Ultra II FS kit). The resulting DNA fragments were sized selected (200 to 500 bp) using AMPure XP beads, the fragments were end repaired, and Illumina-specific adapter sequences were ligated to each fragment. Each sample was individually indexed, and a second size selection step was performed. Samples were then quantified using a fluorometric method, diluted to a standard concentration (4 nM), and sequenced on the Illumina NextSeq platform, using a NextSeq 300-cycle kit, to generate a total of 1,240,880 reads with a 2 × 150-bp paired-end read length. The sequence data were analyzed using specific bioinformatic outfits accessed through the KBase platform v0.11.1 (8). Read processing was performed using Trimmomatic v0.36 (9) and FastQC v0.11.5 for low-quality read filtering and quality control assessment, respectively. The genome assembly was performed with SPAdes v3.13.0 (10). Functional annotation of the whole genome was performed with the NCBI Prokaryotic Genome Annotation Pipeline (PGAP) v4.12 (11). Default parameters were used for all software.

The genome of Campylobacter fetus subsp. *fetus* NWU_ED24 has a total length of 1,801,207 bp assembled in 143 contigs. The assembled genome includes 6,100 coding sequences (CDSs) with a GC content of 33.04%; the *N*_50_ and *L*_50_ values are 31,543 bp and 18, respectively. The genome contains a total of 194 identified subsystems, 2,001 protein-coding sequences, and 36 RNAs. A plethora of putative CDSs for mobile genetic elements and drug resistance genes (e.g., *pgsA* and *gidA*) and multidrug efflux pumps (e.g., *ykkCD*, *macA*, and *macB*) were found. CDSs involved in colonization, adhesion, motility, and invasion were also identified (12).

### Data availability.

This complete genome shotgun project has been deposited in DDBJ/ENA/GenBank under the accession number JACASG000000000. The version described in this paper is version JACASG010000000. The raw reads were submitted with SRA accession number SRX8607532 and BioSample number SAMN15356666.
